# Subclinical myocardial edema and arrhythmic burden in mitral valve prolapse: Insights from T2 mapping

**DOI:** 10.21542/gcsp.2025.25

**Published:** 2025-05-15

**Authors:** Francesco Mangini, Antonio Di Monaco, Luca Sgarra, Roberto Calbi, Francesco Spinelli, Ilaria Dentamaro, Robert W.W. Biederman, Grazia Casavecchia, Matteo Gravina, Massimo Grimaldi

**Affiliations:** 1 Department of Cardiology, Ospedale Regionale “Miulli”, Acquaviva delle Fonti (BA), Italy; 2 Department of Radiology, Ospedale Regionale “Miulli”, Acquaviva delle Fonti (BA), Italy; 3 Department of Cardiology, AOU Policlinico, Bari, Italy; 4 Department of Cardiology, Roper/ St Francis Hospital, Charleston, SC, US; 5 Department of Cardiology, AOU Ospedali Riuniti, Foggia, Italy; 6 Department of Radiology, AOU Ospedali Riuniti, Foggia, Italy

## Abstract

Mitral valve prolapse is a common condition often considered benign; however, its association with arrhythmogenic risks is well-established, particularly when linked with mitral annular disjunction and myocardial fibrosis. Edema often precedes fibrosis in many clinical conditions, and both edema and myocardial fibrosis are detectable in vivo using cardiac magnetic resonance imaging. In cases of mild or chronic edema (subclinical edema), standard T2-weighted sequences may not be sufficient for identification; however, T2 mapping sequences can detect such edema. This study aimed to assess whether subclinical myocardial edema identified through T2 mapping is related to arrhythmic burden in patients with mitral valve prolapse and mitral annular disjunction, even in the absence of fibrosis. Thirty-four patients underwent cardiac magnetic resonance imaging and were classified into low and high arrhythmic burden groups based on the Lown grading system. Inclusion criteria were normal myocardial signal intensity on conventional T2-weighted imaging, non-hemodynamically significant mitral regurgitation, presence of mitral annular disjunction, and absence of fibrosis. The analysis was twofold: segmental, comparing native T2 times between corresponding myocardial segments of the groups; and regional, contrasting basal segments of the lateral, inferolateral, and inferior walls, typically more affected by mitral valve prolapse, with remote myocardium within each group. Patients with higher arrhythmic burdens showed elevated native T2 times, indicating subclinical myocardial edema in the basal myocardial segments. This suggests that even without fibrosis, early myocardial alterations related to mitral valve prolapse may be related to arrhythmic risks. The clinical implications are significant, advocating the integration of T2 mapping into routine evaluations to enhance therapeutic decision making and patient outcomes. The early detection of myocardial changes may allow timely intervention and potentially modify the disease course. Further research is needed to confirm these findings and explore the utility of T2 mapping in managing mitral valve prolapse, even in patients without mitral annular disjunction.

## Introduction

Mitral valve prolapse (MVP) is one of the most common valvular diseases and is often considered benign. However, its arrhythmogenic potential has been widely demonstrated^1^. Among the most critical factors correlating with arrhythmogenesis and the risk of sudden cardiac death, mitral annular disjunction (MAD) and myocardial fibrosis stand out^2,3^. The latter involves up to 42% of patients with MAD^4^ and is typically identified in vivo through cardiac magnetic resonance (CMR) using Late Gadolinium Enhancement (LGE)^5^. Previous studies have shown that altered myocardial wall stress may be associated with myocardial edema^6–8^, a phenomenon that precedes fibrosis development in various cardiac conditions^9^. CMR plays a crucial role in identifying myocardial edema, typically visualized as hyperintensity on T2-weighted sequences^10^. However, in non-acute settings, myocardial edema may be subtle, subclinical, and undetectable using conventional T2-weighted imaging, which would otherwise show a normal myocardial signal. In these cases, T2 mapping sequences provide a valuable tool, allowing the absolute quantification of T2 relaxation times and enabling the detection of subtle differences between myocardial segments^11^. Edema is classically associated with prolonged native T2 values, which may serve as an early marker of myocardial involvement in MVP-related arrhythmogenesis^11,12^.

### Background

MVP is defined as the abnormal systolic displacement of one or both mitral valve leaflets into the left atrium, often leading to mitral regurgitation^13^. In cardiovascular imaging, MVP is defined as systolic displacement of one or both leaflets by at least 2 mm above the mitral annular plane in the sagittal view^5^. The clinical course of patients with MVP is largely influenced by the severity of the associated mitral regurgitation and the presence of arrhythmogenic complications. The key arrhythmogenic risk factors are MAD and myocardial fibrosis^3,14^. As a result, new descriptors such as “arrhythmic” and “malignant” have been introduced to classify this condition, leading to the recognition of a distinct clinical entity: arrhythmic mitral valve prolapse^15,16^.

### Cardiac magnetic resonance

CMR is considered the gold standard for evaluating a series of myocardial diseases, providing high-resolution images of cardiac structures and enabling detailed analysis of ventricular function, volumes, morphologic features (e.g., MAD) and myocardial fibrosis^17^. LGE sequences are widely used to detect myocardial fibrosis, highlighting scar tissue areas that accumulate the contrast agent^5,14,17^. However, the absence of LGE does not exclude the presence of subclinical myocardial alterations, which can be detected using more sensitive methods such as T1 and T2 mapping.

### Myocardial edema and arrhythmias

Myocardial edema is a condition characterized by the accumulation of interstitial fluid in the myocardial tissue and is often associated with inflammation and cellular damage. Myocardial edema has been described in several pathological conditions as well as in some paraphysiological conditions, such as high-intensity physical activity^6^. In patients with MVP, repeated mechanical stress may induce localized inflammation, leading to transient or chronic edema^4,18^. If left untreated, mechanical stress can progress to myocardial fibrosis^19^, increasing the risk of malignant ventricular arrhythmias. T2 mapping allows for the quantification of myocardial edema^20^, providing an early indication of myocardial involvement preceding fibrosis development.

### Clinical implications

Early identification of myocardial edema in MVP patients with a high arrhythmic burden may have important clinical implications, allowing timely interventions to prevent progression to myocardial fibrosis and reduce the risk of malignant arrhythmias. Integrating T2 mapping into the standard evaluation of patients with MVP could improve risk stratification and guide therapeutic decisions, ultimately contributing to better long-term outcomes.

### Objectives

This study aimed to investigate the relationship between native T2 times and left ventricular arrhythmic burden in patients with MVP and MAD, as classified by the Lown grading system.

## Material and Methods

We prospectively enrolled 34 patients diagnosed with MVP and non-hemodynamically significant mitral regurgitation, all of whom were evaluated using CMRI. Each patient exhibited ventricular arrhythmias with a morphology indicative of an MVP-related substrate, and alternative causes of arrhythmias were excluded. The CMRI protocol included assessments of ventricular volume and function, detection of fibrosis via late gadolinium enhancement (LGE) sequences, traditional T2-weighted sequences, and measurement of native T2 times using T2 mapping.

All CMR scans were performed using a 1.5 T scanner (Philips Ingenia Ambition 1.5T X). The protocol included balanced steady-state free precession (bSSFP) sequences with TR/TE ≈ 3.0/1.5 ms, flip angle of 60° , and slice thickness of eight mm. T2-weighted images: short tau inversion recovery (STIR) with TR/TE ≈ 2000/70 ms and slice thickness = 10 mm. T2 mapping: multi-echo bSSFP with three echo times (TEs = 0, 24, and 55 ms) and slice thickness of eight mm. LGE images acquired 10–15 min after gadolinium-based contrast (0.1 mmol/kg) using inversion recovery gradient-echo sequences.

**Figure 1.**
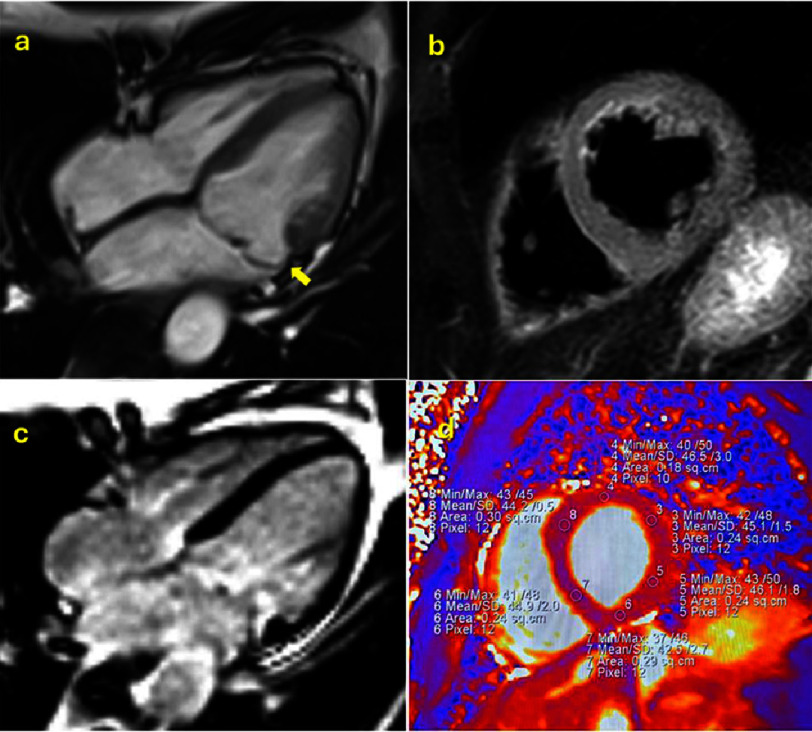
Patients were evaluated by 1.5 T magnetic resonance for volumes, function and morphological study. (a), traditional T2-weighted sequences (b), native T2 mapping (d) and LGE sequences (c); the inclusion criteria were the presence of mitral annulus disjunction (a, yellow arrow), the absence of LGE (c) and the normal myocardial signal in T2-weighted sequences (b) and non-hemodynamically significant mitral regurgitation.

Inclusion criteria were evidence of MAD, non-hemodynamically significant mitral regurgitation defined by regurgitant fraction <30% and absence of LV dilatation or dysfunction, and normal myocardial signal intensity on conventional T2-weighted imaging, defined by a myocardium-to-skeletal muscle signal intensity ratio <2.0. and absence of LGE-detected fibrosis (Figure 1).

Two independent observers re-analyzed T2 maps from 10 randomly selected patients. Intra-observer reproducibility was assessed by repeating measurements after a 2 weeks interval. Inter- and intra-observer agreement was assessed using intraclass correlation coefficients (ICCs), which were 0.86 and 0.89 respectively, indicating good reproducibility.

Patients were stratified into two groups based on their Lown class: 17 patients with a low arrhythmic burden (Lown classes 0–1) and 17 patients with a high arrhythmic burden (Lown classes 2–3–4). Characteristics of both groups are summarized in Table 1. Two types of comparison were made:

**Table 1:** Characteristics of the two groups.

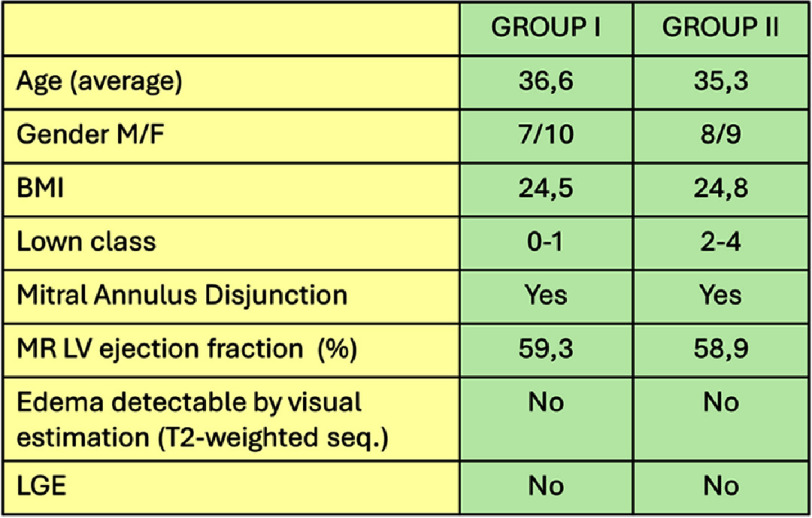

- Segmental analysis: We compared native T2 times between the corresponding myocardial segments in the low and high arrhythmic burden groups.
- Regional analysis: We compared the basal segments of the lateral, inferolateral, and inferior walls, which are typically more mechanically affected by MVP, to the remote myocardium of the septal and anterior walls in each patient in both groups.

### Statistical analysis

We analyzed the data using both parametric and non-parametric tests depending on their distribution. To assess normality, we applied the Shapiro–Wilk test. For comparisons between groups, we used independent samples t-tests when the data were normally distributed, and Mann–Whitney U tests otherwise. Within-group comparisons were performed using paired t-tests or Wilcoxon signed-rank tests depending on the data distribution. To account for multiple comparisons, we applied Bonferroni correction, considering *p* < 0.05, as the threshold for statistical significance. A formal power analysis was not conducted due to the exploratory nature of the study. However, the sample size was based on comparable imaging studies and provided sufficient power to detect group differences in T2 values, with a large effect size (Cohen’s *d* > 0.8).

**Figure 2.**
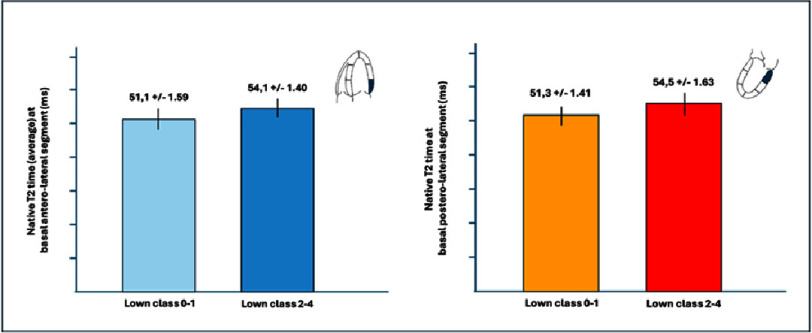
Native T2 values (average) at basal antero-lateral segment (left) and at postero-lateral segment (right) in the two groups.

**Figure 3.**
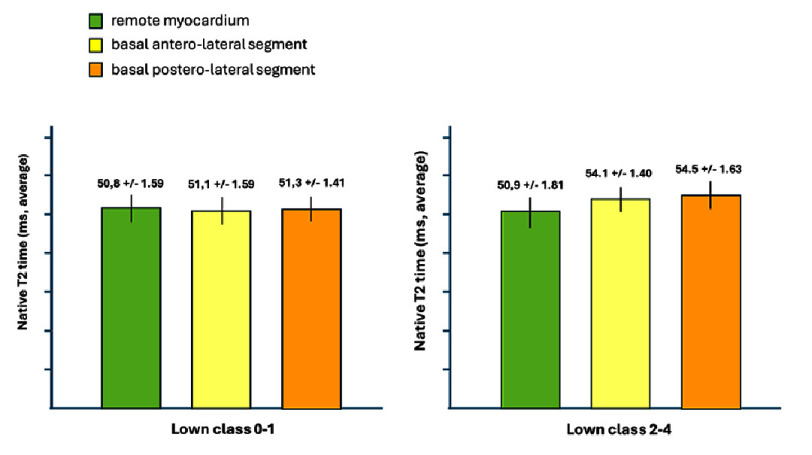
Native T2 values (average) at basal segment of antero-lateral and postero-lateral walls and remote myocardium in the two groups.

## Results

In our analysis, two key comparisons highlighted the impact of the arrhythmic burden on myocardial tissue characteristics as measured by native T2 times. First, patients in the high Lown class group (Lown 2–3–4) exhibited significantly elevated native T2 times in the basal segments of the lateral, inferolateral, and inferior walls when compared to those in the low Lown class group (Lown 0–1). For instance, within the high Lown group, the basal segments of the antero-lateral wall showed a mean native T2 time of 54.1 ± 1.40 ms versus 51.1 ± 1.59 ms in the low Lown group (*p* = 0.027), while the postero-lateral wall measured 54.5 ± 1.63 ms compared to 51.3 ± 1.41 ms (*p* = 0.001) (Figure 2). In the second comparison, we assessed the native T2 times between the basal myocardial segments and remote myocardial regions within the high arrhythmic burden group. Here, the basal antero-lateral segments again demonstrated significantly higher native T2 times (54.1 ± 1.40 ms) compared to remote myocardium (50.9 ± 1.81 ms, *p* = 0.0002). Similarly, the basal postero-lateral segments recorded values of 54.5 ± 1.63 ms versus 50.9 ± 1.81 ms in remote regions (*p* = 0.0001) (Figure 3). Notably, these significant differences between the basal and remote regions were not observed in the low Lown class group.

## Discussion

The relationship between MVP, MAD, myocardial fibrosis, and arrhythmias has been well-documented, with previous studies showing that fibrosis detected by LGE is associated with a higher risk of malignant ventricular arrhythmias^3,4,21–23^. However, a population of patients exhibits complex ventricular arrhythmias even in the absence of documented fibrosis^3^. The findings of this study suggest that patients with MVP and a higher arrhythmic burden exhibit increased native T2 times in myocardial segments that are typically subjected to greater mechanical stress. This suggests the presence of subclinical myocardial edema, which is not detected by conventional T2-weighted sequences but is revealed by T2 mapping^20^.

The association between edema and ventricular arrhythmias has been demonstrated in different pathological settings such as ischemic heart disease and inflammatory myocardial diseases^24^. Therefore, it is conceivable that edema may also play a role in determining arrhythmias in patients with MVP. In contrast, myocardial edema is often a precursor of fibrosis in different settings^7,25^. Thus, these findings may indicate an early pathological stage of the progression from MVP to myocardial scarring and increased arrhythmogenicity.

However, the current study expands on these findings by demonstrating that, even in the absence of LGE-detectable fibrosis, subclinical myocardial alterations can be identified using T2 mapping. The mechanistic link between increased myocardial wall stress and edema formation remains an area of active research. One hypothesis is that repeated mechanical stress from MVP may induce localized myocardial inflammation^26^, leading to transient or chronic edema. In this context, MAD has been shown to be a significant contributor, representing an additional mechanical stress burden^4,21^. MAD has been shown to be associated with increased extracellular volume and edema, as identified by traditional imaging sequences^26,27^. However, the potential for detecting subclinical edema and its possible clinical implications, such as predisposition to arrhythmias, even in the absence of edema on traditional sequences and without fibrosis, has been less explored. Over time, this could evolve into fibrosis, increasing the risk of ventricular arrhythmias. Furthermore, the ability to detect subclinical myocardial edema before fibrosis onset could have profound implications in clinical practice.

Early interventions, whether pharmacological or through lifestyle modifications, could potentially halt or reverse the pathological process, thereby preventing progression to fibrosis and reducing the risk of life-threatening arrhythmias. In clinical settings, the integration of T2 mapping into routine evaluation protocols for patients with MVP could enhance risk stratification and inform personalized therapeutic strategies. For instance, patients with elevated native T2 times might benefit from closer monitoring, more aggressive anti-inflammatory treatments, or other targeted therapies aimed at minimizing myocardial stress and inflammation. Moreover, this approach could pave the way for the development of new therapeutic agents specifically designed to address myocardial edema and its sequelae. As we continue to unravel the complex pathophysiology of MVP-related arrhythmias, insights gained from advanced imaging techniques such as T2 mapping will be invaluable in guiding future research and improving patient outcomes. Confounding factors, such as hydration status, medications, heart rate variability, and loading conditions, may influence T2 values. Although controlled, residual confounding remains possible.

### Limitations

The relatively small sample size and single-center design of the study may limit the generalizability of the findings to broader patient populations. Although LGE was consistently negative, the presence of subtle or diffuse myocardial fibrosis below the detection threshold of LGE could not be entirely excluded. Although the reproducibility of T2 mapping in this cohort was high, further multicenter studies are warranted to confirm these results and strengthen the external validity of the technique.

## Conclusions

Patients with MVP and MAD who exhibit a higher arrhythmic burden show elevated native T2 times in basal myocardial segments, indicating subclinical myocardial edema undetectable by traditional imaging. This early edema may progress to fibrosis and increase the risk of malignant arrhythmias. These findings support the potential of native T2 mapping as a valuable imaging tool for the early detection of myocardial involvement, risk stratification, and guidance of preventive strategies in patients with mitral valve prolapse. Notably, the utility of T2 mapping may extend even to patients with MVP without MAD, offering the ability to detect subtle subclinical myocardial alterations that may contribute to arrhythmogenic vulnerability. The integration of native T2 mapping into the routine evaluation of patients with MVP, particularly those with arrhythmic burden but no detectable fibrosis, could enable more personalized clinical management. Elevated T2 values in this context may serve as early imaging biomarkers of myocardial tissue changes compared with late gadolinium enhancement, thus refining risk assessment and informing closer monitoring or targeted therapeutic strategies. Further prospective studies are warranted to validate these preliminary findings, clarify their prognostic implications, and explore their impact on the long-term outcomes in this patient population.

## Conflicts of interest

All authors declare no conflict of interest.
